# A sticky situation: When trypanosomatids attach to insect tissues

**DOI:** 10.1371/journal.ppat.1011854

**Published:** 2023-12-21

**Authors:** Megan L. Povelones, Nikki A. Holmes, Michael Povelones

**Affiliations:** 1 Department of Biology, Villanova University, Villanova, Pennsylvania, United States of America; 2 Department of Pathobiology, University of Pennsylvania School of Veterinary Medicine, Philadelphia, Pennsylvania, United States of America; Joan and Sanford I Weill Medical College of Cornell University, UNITED STATES

## Abstract

Transmission of trypanosomatids to their mammalian hosts requires a complex series of developmental transitions in their insect vectors, including stable attachment to an insect tissue. While there are many ultrastructural descriptions of attached cells, we know little about the signaling events and molecular mechanisms involved in this process. Each trypanosomatid species attaches to a specific tissue in the insect at a particular stage of its life cycle. Attachment is mediated by the flagellum, which is modified to accommodate a filament-rich plaque within an expanded region of the flagellar membrane. Attachment immediately precedes differentiation to the mammal-infectious stage and in some cases a direct mechanistic link has been demonstrated. In this review, we summarize the current state of knowledge of trypanosomatid attachment in insects, including structure, function, signaling, candidate molecules, and changes in gene expression. We also highlight remaining questions about this process and how the field is poised to address them through modern approaches.

## Introduction

Trypanosomatids are a group of flagellated eukaryotic parasites (class Kinetoplastea) that can infect plants, mammals, and invertebrate hosts [1]. Most trypanosomatids are monoxenous and only infect insects [2], while dixenous parasites use insects as both hosts and vectors to infect other hosts. Therefore, successful colonization of the insect is crucial for completion of almost all trypanosomatid life cycles [3,4]. Despite this, less is known about developmental transitions in the insect, as most studies have focused on mammalian forms. As several of the dixenous trypanosomatids cause important human and animal diseases, increased understanding of these transitions in the insect vector could lead to new ways of blocking their transmission. Within this context, addressing mechanisms shared by all pathogenic species is particularly attractive [5].

**Fig 1:**
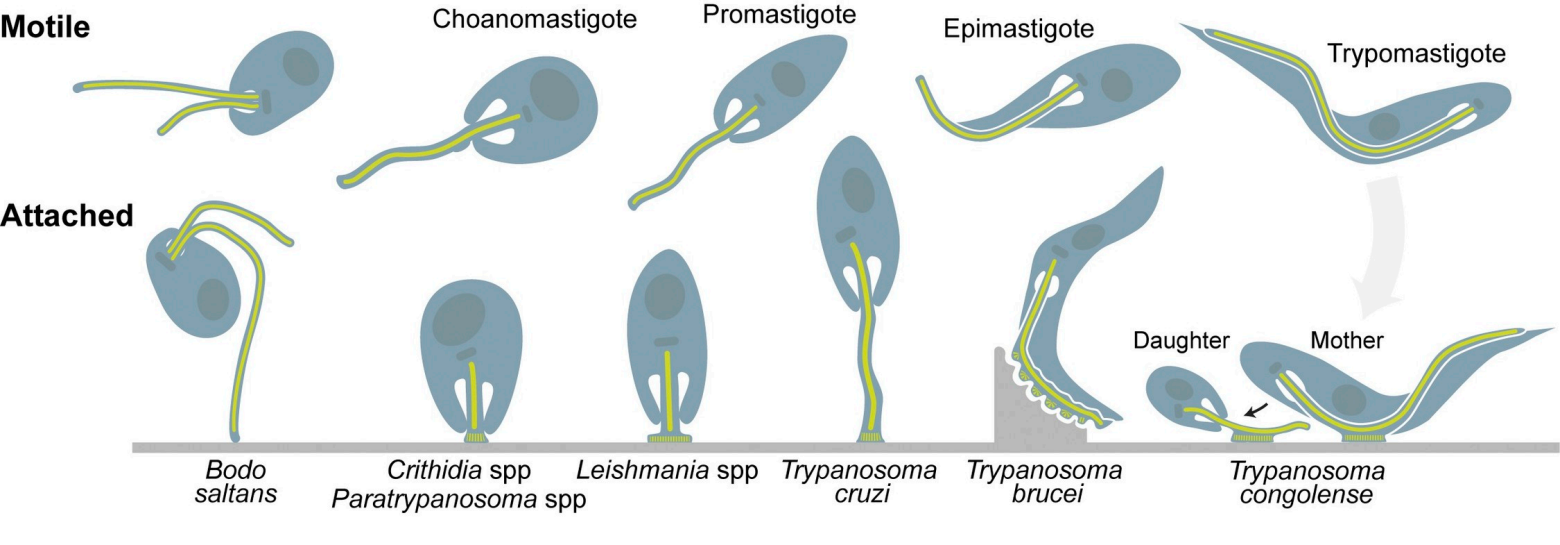
Diagrams of trypanosomatids in their motile and attached forms. The large dark oval is the nucleus. The small dark disk is the kDNA. Green is the axoneme. Attachment plaques are shown as green lines in attached forms. A detailed description of swimming morphologies can be found in [15]. In monoxenous parasites and *Leishmania* spp., the attachment plaque forms at the tip of the shortened flagellum. In *T*. *cruzi*, the attachment plaque is also found at the tip but the flagellum is only slightly shorter. In *T*. *brucei* and *T*. *congolense*, the attachment plaque(s) form on the lateral portion of the flagellum.

Parasites undergo developmental transitions to adapt to distinct environmental niches and to allow transmission between hosts [6–9]. In trypanosomatids, these forms are named according to the length and position of the cell’s single flagellum [10–13]. The flagellum emerges from an invagination of the plasma membrane called a flagellar pocket. In species such as *Trypanosoma cruzi*, *T*. *brucei*, and *T*. *congolense*, the flagellum is attached (juxtaform) along the length of the cell body by an undulating membrane, extending as a free flagellum just beyond the anterior end of the cell. In *Leishmania* spp. as well as in monoxenous species, the flagellum is unattached (liberform) [14,15]. In both cases, the cell swims towards its flagellum (Fig 1) [16,17]. At the base of the flagellum is the basal body, which in addition to assembling the microtubules of the axoneme, is central for cellular organization including segregation of the mitochondrial DNA network (kinetoplast DNA or kDNA) [18,19]. While most developmental forms have an active flagellum and are motile, sessile forms attached to insect host tissues are also observed and are sometimes called haptomonads [20–22]. Attachment, also referred to as adherence or adhesion, is a conserved process mediated by the flagellum [23]. In this review, we will use the terms “adhesion” or “adherence” to refer to a transient association between the parasite flagellum and a host tissue that occurs without overt structural alterations. The term “attachment” will be used to indicate when parasites are stably attached to insect tissues via a modified flagellum, including a hemidesmosome-like attachment plaque.

Although first described decades ago (reviewed in [23]), many questions remain regarding the mechanisms and functions of attachment (Box 1). However, recent studies using in vitro approaches, advanced imaging, proteomics, and single-cell RNA sequencing have provided new insights into this critical process. In this review, we aim to summarize our current understanding of trypanosomatid attachment and outline exciting possibilities for future research.

**Box 1. Major questions about trypanosomatid attachment**

- Why do trypanosomatids attach to specific insect tissues?
- What are the parasite determinants of adhesion and attachment?
- Are there signals/molecules in the vector host that impact attachment?
- What factors determine the number/location of flagellar attachments?
- What signaling pathways control differentiation between motile and attached states?
- What molecules comprise the hemidesmosome-like attachment plaque and how are they localized?

### The role of attachment in the life cycle

Trypanosomatids evolved from free-living species called bodonids [24]. Bodonids have 2 structurally distinct flagella extending from the anterior end of the cell and which are named anterior and posterior (or recurrent) based on their position relative to each other [25]. Species such as *Bodo saltans* can attach to surfaces via the tip of their posterior flagellum, using their more motile, anterior flagellum to sweep bacteria into their cytostome for internalization and digestion [25,26]. Similarly, free-living trypanoplasms such as *Procryptobia glutinosa* appear to have 2 forms: a free-swimming form and a form that creeps along the substratum using its posterior flagellum to skid across the surface [27]. Therefore, adhesion to substrates via the flagellum may be an ancestral trait that predates the evolution of parasitism within this group. Some parasitic bodonids, such as the biflagellate *Cryptobia vaginalis*, also glide along surfaces on their posterior flagellum [28]. However, there are important differences between surface adhesion by bodonids and trypanosomatids. First, while bodonids use their posterior flagellum for adhesion, it is the anterior flagellum that has been retained by the trypanosomatids [29]. Second, an attachment plaque stably connecting the bodonid flagellum to a surface has not been reported [30].

Among the parasitic trypanosomatids, monoxenous lifestyles likely predate dixenous life cycles [2]. Monoxenous parasites are transmitted when an infected insect defecates onto a food source, releasing parasites that can survive for a brief period before being ingested by another insect. Ingested parasites migrate and attach to the hindgut and rectum via their flagellum, although they are sometimes found in other tissues [31]. At the point of attachment, the flagellar membrane expands to accommodate a filamentous structure called an attachment plaque that resembles a mammalian hemidesmosome. Heavy infections feature dense layers of parasites covering the entire surface of affected tissues. Each parasite is independently attached via its flagellum which, probably due to crowding, varies in length according to the distance between the parasite and the surface [32]. While binary fission of attached cells allows for colonization of insect tissue, transition to motile, detached forms is required to infect new hosts.

*T*. *cruzi* attaches to the gut of a triatomine bug [33]. Ingested trypomastigotes differentiate to replicative epimastigotes in the midgut. Epimastigotes are defined by a cellular rearrangement in which the kDNA-basal body complex migrates from a posterior to anterior position relative to the nucleus [10]. Interestingly, this configuration is shared by attached forms of all trypanosomatids, although only those with a juxtaform flagellum shift their organelles to achieve it (Fig 1). Epimastigotes adhere via their flagellum to the triatomine posterior midgut before attaching to the hindgut and rectum. Attached parasites differentiate to motile, infectious metacyclics that will be excreted when the insect takes its next blood meal, entering the mammalian host through the wound or mucosa [34].

*Leishmania* amastigotes enter the sandfly when it ingests infected macrophages during a blood meal [20]. Amastigotes differentiate into procyclic promastigotes, which replicate in the midgut before becoming long nectomonad promastigotes capable of escaping the peritrophic matrix created by the fly during bloodmeal digestion. Long nectomonads adhere to the midgut epithelium via their flagella, which insert between the microvilli of the gut cells, preventing defecation of the parasites with the remnants of the blood meal. Long nectomonads then de-adhere from the midgut, become short nectomonads (or leptomonads), and migrate to the anterior portion of the gut. Here, they attach to the stomodeal valve, becoming sessile haptomonads and eventually differentiating to infectious motile metacyclics. The attached haptomonads secrete a chitinase [35] as well as a gel-like substance composed of proteophosphoglycans [36] that compromise valve function and increase the likelihood of regurgitation during blood feeding, thus facilitating transmission [37].

*T*. *cruzi* and *Leishmania* display both flagellar adherence and attachment during their life cycles [38,39]. First, parasites adhere to the epithelial cells or perimicrovillar membranes (PMMs) lining the midgut [40–43]. The flagellum interdigitates between microvilli, but structural modifications to the flagellum are not evident. Later, parasites stably attach to a cuticular surface: the hindgut and rectum for *T*. *cruzi* and the stomodeal valve for *Leishmania* (although *Leishmania* species of the subgenus *Viannia* or *Sauroleishmania* can also colonize the hindgut as attached haptomonads before returning to the midgut [20]). The cuticle consists of chitin overlayed with a waxy, lipid-rich coating. It is to this superficial, hydrophobic surface that parasites attach [44,45]. This attachment precedes differentiation to infectious forms (metacyclics) and features a highly modified flagellum including a filament-rich attachment pad and an expanded membrane [38,39]. Both adherence and attachment prevent premature elimination of the parasites. Specifically, cuticular attachment may block the release of metacyclics until development is complete.

*T*. *brucei*, including human-infective subspecies, enter their tsetse vector and replicate in the midgut and ectoperitrophic space as procyclic form parasites. They then migrate anteriorly, pass through the proventriculus, and reach the salivary gland. During this migration, parasites undergo an asymmetric division event, producing a long and short epimastigote [46,47]. The short epimastigote attaches to the salivary gland epithelium and can divide to produce more attached cells [47,48]. Unlike *T*. *cruzi* and *Leishmania* parasites, *T*. *brucei* attach directly to epithelial cells via flagellar attachment plaques [49]. Some of these parasites differentiate into unattached, non-replicative metacyclics that can be transmitted by the fly to a mammalian host.

The animal pathogens *T*. *congolense* and *T*. *vivax* both attach to the proboscis and labrum of the tsetse fly [50,51]. Similar to *T*. *brucei*, *T*. *congolense* replicates in the midgut before migrating to the proventriculus and eventually the mouth parts [52]. Here, parasites attach to the cuticular lining of these tissues and replicate as attached cells. In contrast, *T*. *vivax* immediately attaches to the fly mouth parts, divides, and develops infectious forms without entering the midgut [53]. For both *T*. *congolense* and *T*. *vivax*, attached epimastigotes give rise to free-swimming metacyclics, which are infectious to the next host [54,55].

### The initial stages of attachment

**Fig 2:**
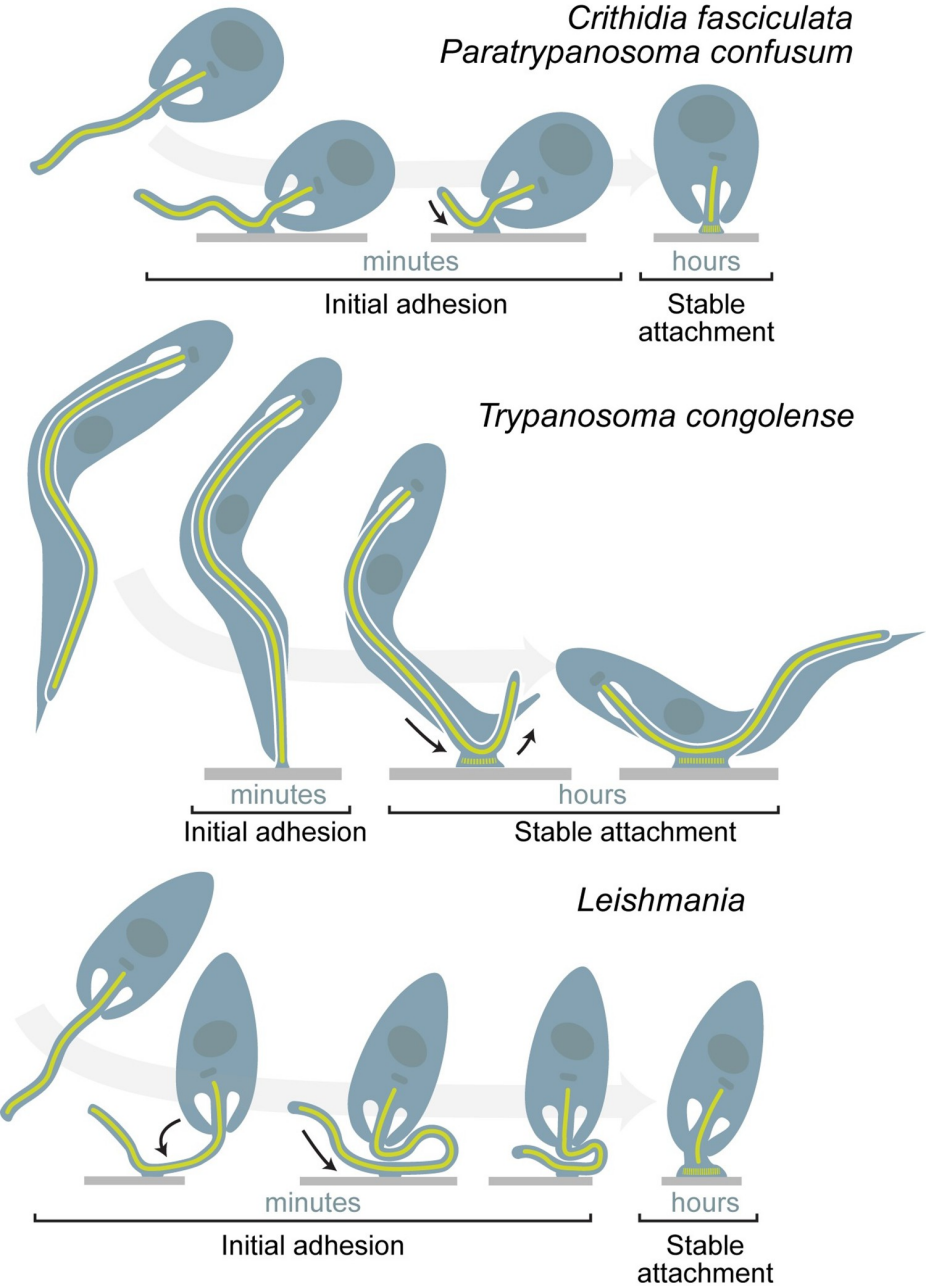
Stages of attachment visualized using in vitro systems. *C*. *fasciculata* and *P*. *confusum* attach via the base of the flagellum, which then shortens until the cell rests perpendicular to the surface on the flagellar attachment plaque [21,57]. *T*. *congolense* trypomastigotes initially attach via the tip of the flagellum. The cell body and flagellum then slide until the cell is resting on the surface connected by a lateral flagellar attachment plaque [62]. *L*. *mexicana* can attach via the tip or lateral (shown) portion of the flagellum. A loop sometimes forms between the point of attachment and the cell body. The flagellum then shortens until the haptomonad rests perpendicular to the surface on the flagellar attachment plaque [56].

Although trypanosomatids encounter many surfaces in the insect, the transition from motile to attached occurs at a particular place and time. Whether this is driven by the developmental stage of the parasite, surface properties, or a combination of these is not understood. To address this rapid transition in a time-resolved manner, organisms that attach to artificial substrates such as plastic and glass are used as model systems (Fig 2). With some variation, attachment is associated with altered flagellar dynamics, cytoskeletal remodeling, and finally formation of an attachment plaque that is virtually identical to that observed in vivo [32,56–60].

In 2 monoxenous parasites of mosquitoes, *Crithidia fasciculata* and *Paratrypanosoma confusum*, motile parasites initially attach to artificial surfaces in vitro via the base of their flagellum [21,61]. In some cases, a visible bulge is present at the point of initial attachment, which then expands to form the haptomonad attachment pad [21,22,59]. Attachment triggers dramatic flagellar shortening until the parasite cell body rests perpendicular to the substrate (Fig 2). The shortened flagellum is remodeled to form an attachment plaque within an enlarged flagellar membrane. Haptomonads are often rounder than swimming forms, suggesting attachment involves additional cytoskeletal rearrangements.

*Leishmania* parasites can also attach to artificial surfaces [45,56] and detailed stages of attachment in vitro have been defined [56]. Initial attachment can occur either at the base of the flagellum or laterally at a position along the flagellum’s length (Fig 2). In both cases, the flagellum shortens and the attachment plaque forms at the tip, anchoring parasites to the substrate. As with their monoxenous cousins, haptomonads are shorter and rounder than swimming promastigotes.

Stable attachment of *T*. *congolense* and *T*. *vivax* occurs along the flagellum’s length [50,58]. In vitro studies in *T*. *congolense* showed that trypomastigotes initially attach at the tip of their flagellum. This attachment later moves to a position along the length of the flagellum that is associated with accumulation of the paraflagellar rod protein PFR1 [62]. The flagellum first lengthens, then shortens by approximately 30% in attached cells as the cell becomes shorter and rounder. While *T*. *brucei* do not attach in vitro, localization of intraflagellar transport proteins indicates that the pre-attached short epimastigote [47] has a more dynamic flagellum compared to other developmental forms [63].

For *T*. *congolense* and *C*. *fasciculata*, attachment can be prevented by shaking the tissue culture flask, supporting the idea that sustained contact with a compatible substrate is sufficient to drive differentiation to the attached form [54,64]. However, attachment can also be impacted by environmental cues. Attachment of *T*. *congolense* is increased in minimal medium lacking serum [65]. Similarly, in vitro attachment of *T*. *cruzi* epimastigotes can be induced by nutrient deprivation in triatomine artificial urine (TAU) medium supplemented with amino acids [66–68]. Nutrient availability can also trigger attachment of non-parasitic diplonemids, leading some to suggest that this property is ancestral in euglenozoa [69]. In *T*. *cruzi*, attachment is again associated with flagellar dynamics, as deprivation of glucose causes the *T*. *cruzi* flagellum to lengthen [70], while attached cells have a shorter flagellum [71]. Starvation conditions may reflect nutrient availabilities in different compartments of the insect, thereby signaling cells to attach [33,72].

### Involvement of surface proteins in attachment

Parasite surface proteins are present at the host–pathogen interface and are clear candidates for attachment factors [73] (Table 1). Trypanosomatids are coated with glycosylated proteins anchored to the membrane by a glycosylphosphatidylinositol (GPI) anchor. GPI-anchored proteins vary between species and between developmental forms of the same species [74]. In the mammalian bloodstream, *T*. *brucei* are covered by GPI-anchored variant surface glycoprotein (VSG). Upon ingestion by a tsetse fly, the VSG coat is replaced by a procyclin coat [75]. Later, the surface protein *Tb*BARP (*brucei* alanine rich protein), a marker for *T*. *brucei* epimastigotes, is found on cells attached to the salivary gland epithelium in the tsetse fly, but it is unknown whether it plays a direct role in attachment [76]. Efforts to identify new GPI-anchored surface proteins revealed another apparently epimastigote-specific family of proteins called *Tb*SGE1 [77,78]. Recently, these proteins have been renamed metacyclic invariant surface protein (*Tb*MISP) after their expression was detected in all salivary gland stages but most highly in the metacyclic form [79]. This discrepancy may be due to the inaccessibility of *Tb*MISP epitopes to antibody-based detection within the dense metacyclic VSG (mVSG) coat, which is acquired in the fly prior to transmission in the mammal. *Tb*MISPs contain a large extracellular domain and many of the same functional regions as the *T*. *congolense* protein *Tcon*CESP (discussed below), suggesting a possible role in attachment. *Tb*MISP isoforms have C-terminal extensions that differ in length, possibly allowing for different types or degrees of attachment [79]. These C-terminal regions also have basic residues that would be susceptible to proteolysis, pointing to a possible mechanism for release of attached cells. Alternatively, it has been suggested that the dense packing of mVSG proteins on the surface during the epimastigote to metacyclic transition in the fly could facilitate detachment required for transmission [49]. *Tb*MISP is not required for completion of the life cycle in the tsetse fly, although since it is not known whether attachment itself is essential (see further discussion of this below), the exact role of *Tb*MISP is still unclear.

**Table 1:** Gene products with possible roles in trypanosomatid adhesion and attachment.

Protein	Organism(s)	Proposed role	Localization	Reference(s)
Surface molecules				
BARP and MISP	*T*. *brucei*	Surface proteins enriched on salivary gland stages	Surface	[76,78,79]
CESP	*T*. *congolense*	Promotes attachment to plastic	Surface	[80]
LPG	*Leishmania*	Midgut adherence	Surface, including flagellum	[81]
FLAG1/SMP1	*Leishmania*	Midgut adherence	Flagellar surface	[82]
Heparin-binding proteins	*Leishmania*, *T*. *cruzi*	Midgut adherence	Surface, including flagellum	[84,85]
GP72	*T*. *cruzi*	FAZ component, attachment, survival in vector	Surface (enriched near posterior end of flagellum)	[151,157,166]
SMUG-s/SMUG-L	*T*. *cruzi*	Attachment/Midgut adherence	Surface	[167]
GIPLs	*T*. *cruzi*	Midgut adherence	Surface	[86]
GP35/50	*T*. *cruzi*	Attachment	Surface (patchy)	[94]
GP63	*C*. *fasciculata*, *Herpetomonas*, *T*. *cruzi*	Attachment/adherence/metacyclogenesis	Surface	[87,98]
Cruzipain	*T*. *cruzi*	Adherence/metacyclogenesis	Golgi/reservosome	[88]
Calpain	*T*. *cruzi*	Adherence/metacyclogenesis	Surface	[89]
Small calpain-related proteins 1–4 and 1–5	*T*. *brucei*	Expressed by salivary gland stages	Surface, including flagellum (Tb927.1.2160, TrypTag)	[78,162,163,168]
KIAP1, 2, and 3	*L*. *mexicana*	Attachment	Attachment plaque	[141]
DGF-1	*P*. *confusum*, *T*. *cruzi*	Differentiation/host interaction	Intracellular/secreted	[21,158]
Galectin (Galec)	*P*. *papatasi*	Adhesion receptor on midgut cells, interacts with LPG	Luminal surface	[169]
LuloG	*Lu*. *longipalpis*	Adhesion receptor on midgut cells	Luminal surface	[170]
Signaling proteins				
ACs	*T*. *brucei*, *T*. *cruzi*	Social motility, differentiation	Flagellum/contractile vacuole	[119,131]
PDEs	*T*. *brucei*, *C*. *fasciculata*, *T*. *cruzi*	Social motility, differentiation	Flagellum	[57,61,171,172]
FLAM8/CARP3	*T*. *brucei*	Social motility, extravasation	Flagellum	[128,131–133]
PAD	*T*. *brucei*, *T*. *cruzi*	Transporter of differentiation signals	Surface/flagellar pocket	[151,155,163]
RNA-binding proteins				
ZC3H31	*T*. *cruzi*	Differentiation	*Tb* ortholog (Tb927.10.5150) is endocytic (TrypTag)	[150,168]
RBP35	*T*. *cruzi*	Differentiation	*Tb* ortholog (Tb927.9.12360) is cytoplasmic (TrypTag)	[151,168]
RBP6	*T*. *brucei*	Differentiation	Cytoplasm/starvation granules	[149,173]
ZC3H11 and ZC3H45	*T*. *brucei*	Differentiation	Cytoplasm (TrypTag)	[78,162,163,168]

Candidate attachment proteins have also been studied in *T*. *congolense* [58,80]. Extraction of attached cells with detergent and high salt removed the cell membrane, PFR, and cytoskeleton but left an electron dense fibrous plaque attached to the substrate. Gel electrophoretic analysis of this material revealed that it was highly and selectively enriched in an approximately 70 kDa protein that has yet to be identified [58]. In another study, supernatant conditioned by epimastigotes, but not procyclic form cells, contains a factor called *Tcon*CESP (*congolense* epimastigote-specific protein), which has multiple predicted N-glycosylation sites as well as a predicted GPI anchor attachment site [80]. *Tcon*CESP has limited similarity to *Tb*BARP and can increase the rate of attachment to plastic. However, in one in vitro study, attachment and differentiation of *T*. *congolense* induced by serum deprivation occurred in the absence of detectable *Tcon*CESP expression [65].

Several surface molecules have been implicated in adhesion of *T*. *cruzi* and *Leishmania* to the midgut of their respective vectors. These include lipophosphoglycan (LPG) [81] and SMP1 [82] in *Leishmania* and heparin-binding proteins in both species [83–85]. In *T*. *cruzi*, glycoinositolphospholipids (GIPLs) [86] and surface proteases such as GP63 [87], cruzipain [88], and calpain [89] all have proposed roles in adherence (see Table 1). While this work is outside the scope of this review, it is interesting to note that interactions between parasites and the midgut epithelium or PMM may be liganded and dependent on the glycosylation state of surface molecules, which is developmentally regulated [90–92].

The most abundant proteins on the surface of *T*. *cruzi* are mucins [93]. Overexpression of *Tc*GP35/50 mucins, surface proteins encoded by the *Tc*SMUG-S family of genes, specifically increased attachment to the rectal tissue of *T*. *infestans* [94]. *Tc*GP35/50 proteins are found on the entire surface, not just on the flagellum, so it is not clear if this is the primary mediator of attachment. However, competitive binding assays support involvement of the smugS peptide and a branched Beta-Gal*f* trisaccharide in attachment, although this may be strain specific [94].

GP63s are GPI-anchored surface metalloproteases that are developmentally regulated [95]. These proteins, also called leishmanolysins, are present or even expanded in related free-living members of euglenozoa [69]. Anti-GP63 antibodies and divalent metal chelators decreased *Leishmania* adherence to dissected midguts in vitro [96] but are dispensable for development in the sandfly [97]. However, numerous studies have linked GP63s to attachment by monoxenous species [61,98,99]. Although their exact function is unknown, surface proteases could provide direct ligands, expose ligands through proteolysis, or remodel the substrate to favor attachment.

### Attachment-related signal transduction

Differentiation events are controlled by signal transduction pathways [100,101]. In the context of attachment, signaling may allow parasites to sense their environment and attach to an appropriate surface in the insect. Reinforcing signals might direct the transition from initially to stably attached, including formation of the attachment plaque and associated cytoskeletal remodeling. Finally, intrinsic or extrinsic factors could direct parasites to detach and differentiate to free-swimming forms.

One mechanism for environmental sensing is through changes in metabolic flux. Nutrient depletion increases the efficiency of *T*. *cruzi* attachment and drives differentiation to the metacyclic form in both *T*. *cruzi* [102] and *Leishmania* [92]. In both species, decreased metabolic flux may trigger compensatory increases in endocytosis and autophagy [68,103]. In *T*. *cruzi*, endocytosed material is stored in organelles called reservosomes, which can supply nutrients for metacyclogenesis [104]. Surface proteases such as those discussed above may serve a nutritional role, creating small peptides for endosomal uptake and delivery to the lysosome or proteasome for further digestion. Supporting this, proteasome inhibitors blocked metacyclogenesis in *T*. *cruzi*, although attachment was unaffected [105,106]. An increased endocytic rate would also facilitate surface coat turnover, possibly driving the transition from attached to motile forms.

The highly diffusible second messenger cyclic AMP (cAMP) is involved in differentiation-related signal transduction in trypanosomatids [107,108]. cAMP is produced by adenylate cyclases (ACs) and degraded by phosphodiesterases (PDEs), which convert cAMP into 5′-AMP. ACs in trypanosomatids are mostly transmembrane proteins with a potential ligand-binding extracellular domain, although thus far no ligands have been identified. ACs are activated by dimerization [109,110], which may depend on ligand binding [111].

Attachment-related signal transduction in *T*. *cruzi* involves 2 transient cAMP peaks: one during nutrient deprivation and a second after attachment ([112] and reviewed in [100]). Supporting the role of this pathway in the epimastigote to metacyclic transition, differentiation can be triggered by cAMP analogs and PDE inhibitors [113,114]. In one model, the high osmolarity of both TAU culture medium and the triatomine hindgut causes the cell to swell, activating a mechanosensitive channel in the contractile vacuole (CVC) [115]. This triggers regulatory volume decrease including release of uncharged or acidic amino acids and an increase in intracellular calcium, possibly activating calcium-sensitive ACs [116,117]. A PDE localized to the CVC could terminate the signal [118]. Recent work on *T*. *cruzi* AC1 and AC2 found that they are present at both the flagellar tip and the CVC [119]. When *Tc*AC1 is overexpressed, it increases both attachment and metacyclogenesis, an effect that can be rescued by expression of truncated mutants with altered localization. These truncations also reduced the parasites’ ability to recover from hyperosmotic stress through regulatory volume decrease [119].

Transcripts for predicted cAMP signaling proteins are differentially regulated in motile compared to attached *C*. *fasciculata* [61]. PDE inhibitors prevent attachment in vitro but have no effect on the growth rate of either motile or stably attached cells [57]. We conclude that a transient, PDE-mediated drop in cAMP is required for the developmental transition from motile to attached but is not required for maintenance of either developmental form.

cAMP signaling is required for social motility (SoMo) of *T*. *brucei*, a form of collective movement (reviewed in [120]). When cAMP signaling proteins are reduced or inhibited, parasites are motile and viable but remain in the midgut of their tsetse vector [120]. Since these parasites never reach the salivary glands, and *T*. *brucei* does not attach in vitro, it is unknown whether parasites with impaired cAMP signaling also fail to attach. This question might be addressed in *T*. *congolense* using PDE inhibitors or newly developed genomic techniques paired with in vitro attachment assays [121]. On a shorter time scale, swarms of *T*. *brucei* cells in tsetse compartments show synchronous flagellar beating, possibly driven by hydrodynamic forces, although flagella of attached cells did not beat synchronously [122]. While cAMP signaling has been showed to impact the flagellar waveform of individual *Leishmania* parasites [123], it is not known whether this pathway controls the synchronization of flagellar movements.

Intriguingly, proteins involved in cAMP signaling, including ACs [119,124,125], PDEs [126,127], and trypanosomatid-specific cAMP response proteins (CARPs) [128] are localized to the flagellum which is also the site of attachment. In fact, while some of these proteins localize throughout the flagellum, others localize to subdomains such as the flagellar tip, suggesting that cAMP signaling may be spatially regulated in structured microdomains [119,124,129]. The localization of cAMP signaling proteins can differ in motile versus attached cells, perhaps as a result of flagellar remodeling [57,112].

Differential expression or localization of cAMP signaling proteins may alter cells’ receptiveness to environmental cues. Surface contact and initial attachment could also impact the distribution or activity of flagellar signaling components through changes in intraflagellar transport. As discussed above, altered flagellar dynamics is a common feature of attached cells, and some cAMP signaling components have been shown to stably associate with structures in the flagellum [116,130,131]. One of these flagellar anchoring proteins is *Tb*FLAM8, located at the flagellar tip in procyclic *T*. *brucei* and throughout the flagellum in bloodstream form [132]. *Tb*FLAM8 is required for SoMo in vitro and completion of the life cycle in the fly [131] and has recently been shown to impact migration of *T*. *brucei* from the mammalian vascular system into the tissue spaces, a process that is not understood but which may involve adhesion to vascular endothelial cells [133,134].

### Structure of the attachment plaque

Ultrastructural analysis of the attachment plaque reveals a remarkably similar structure among trypanosomatids [23]. The point of attachment has 2 layers of electron dense material within the expanded flagellar membrane, which is separated from the substrate by a gap that ranges between 10 and 20 nm and where fibrous material is sometimes observed [23,39,56]. From the plaque, a dense set of filaments of unknown composition extends into the flagellar matrix. These plaques resemble hemidesmosomes that connect mammalian cells to the basal lamina [135,136] and are remarkably stable even in the presence of detergents and proteases [21,58,62]. Strikingly, this attachment is in some cases reversible [61], allowing release of motile parasites to infect their next host.

In a series of papers, Brooker described “hemidesmosomes” connecting the flagellum of *C*. *fasciculata* to the mosquito hindgut/artificial surfaces, as well as “desmosomes” linking the flagellum to the parasite cell body [32,59]. Several investigators have noted that these 2 structures appear connected [50,56,137]. The desmosome-like structure was subsequently renamed the flagellar attachment zone, or FAZ, and consists of a series of junctional complexes connecting the flagellum along the length of the cell body, producing the attached flagellum characteristic of juxtaform species [12,138–140]. In liberform parasites such as *C*. *fasciculata* and *Leishmania*, the FAZ connects the flagellum to the cell body at the neck of the flagellar pocket and is essential for proper configuration of this region. In haptomonad forms, FAZ structures are more numerous and extensive, leading some authors to suggest that the FAZ evolved specifically for the attached stage and was later expanded in species with juxtaform flagella [21].

In almost all cases, the attachment plaque forms between the flagellum and a cuticular surface in the insect, while adhesion to epithelial layers in the midgut occurs without flagellar modifications. In contrast, the flagellum of *T*. *brucei* epimastigotes intercalates in the microvilli of the salivary gland epithelial layer, forming multiple attachment plaques along its length within an expanded flagellar membrane [137]. These plaques form preferentially at the tips of the microvilli, and freeze-fracture experiments revealed arrays of membranous bodies at the host cell membrane at sites of attachment [49]. We speculate that this unusual mode of *T*. *brucei* attachment precludes its ability to attach in vitro.

A recent study describes the structure of attached *Leishmania* haptomonads in detail [56]. The authors used serial block face scanning electron microscopy to create 3D representations of *L*. *mexicana* haptomonads attached to the stomodeal valve. Each parasite was indeed attached to the chitinous outer layer of the valve by an attachment plaque typically found at the tip of the flagellum, although connections between the lateral side of the flagellum and the valve were also observed. Attached parasites were shorter and wider, with shortened flagella, although flagellar length varied with distance between the cell and the surface as has been seen for *C*. *fasciculata* [32]. Whether variation in flagellar length represents parasites in the process of differentiation between motile and attached or cells that extend their flagellum due to crowding of haptomonads is unclear. Dividing haptomonads, in which both daughters were connected to the substrate via individual attachment plaques, were also observed [56].

Excitingly, 3 attachment plaque proteins have recently been described in *L*. *mexicana* [141]. These KIAPs (kinetoplast-insect adhesion proteins) localize to the attachment plaque membrane (KIAP1 and 3) or to the filaments (KIAP2) and are required for attachment both in vitro and in vivo. While KIAP1 is absent from the *Trypanosoma* lineages, KIAP2 and 3 are broadly conserved. Interestingly, no KIAP orthologs were detected in *B*. *saltans*. In swimming promastigotes, KIAP2 is found primarily at the base of the flagellum where it exits the cell body, while KIAP1 and 3 localize diffusely. During initial adhesion, all 3 KIAPs accumulate at points of membrane deformation and stably associate with the attachment plaque during and after flagellar disassembly [141].

### Cell division and regulation of gene expression in attached cells

Trypanosomatids can divide as attached cells, allowing them to colonize insect tissues [49,142,143]. However, division of these attached cells can also produce motile cells required for transmission to the next host. In time-lapse studies of *C*. *fasciculata* attached in vitro, division events seem symmetrical, producing either 2 daughter cells that remain attached or 2 daughter cells that detach and swim away [61].

Asymmetric divisions are also common in trypanosomatid life cycles and are associated with developmental changes in morphology including reductions in flagellar length [144,145]. *T*. *brucei* cells undergo their first asymmetric division during their anterior migration from the midgut to the proventriculus. This division produces a long and a short epimastigote, which attaches to the salivary gland epithelium. Attached *T*. *brucei* epimastigotes undergo 2 types of division [48]. The first is symmetrical, resulting in 2 daughter epimastigote cells. Later in the infection, *T*. *brucei* cells divide asymmetrically, producing 1 epimastigote cell that remains attached and 1 pre-metacyclic cell. The daughter flagella are also asymmetrical, as the new (metacyclic) flagellum is enriched for a calcium-binding protein called calflagin, which is important for infectivity [146], as well as *Tb*FLAM8 [132]. These 2 modes of cell division likely facilitate transmission over the life span of the fly, maintaining large numbers of parasites in the salivary gland that can continually produce infectious metacyclics [48]. Similar findings were made in *T*. *vivax* [53] and *T*. *cruzi*, where evidence suggests that asymmetrical division produces metacyclics or intermediate forms that are more likely to detach [71,142]. The signals that determine whether division is symmetrical or asymmetrical are not known, although physical contact between attached parasites, quorum sensing, or environmental changes due to blood feeding have all been proposed as possible drivers of asymmetrical division and metacyclogenesis.

In trypanosomatids, almost all gene regulation is posttranscriptional (reviewed in [147]). As such, RNA-binding proteins (RBPs) play a significant role in regulating gene expression by stabilizing or destabilizing transcripts during life cycle transitions [148]. A striking illustration of this is that in vitro overexpression of a single *T*. *brucei* RNA-binding protein, *Tb*RBP6, was able to induce a series of developmental transitions that mimic those found in the fly, culminating in the production of metacyclics [149]. It is not mentioned whether in vitro-generated epimastigote forms attach to the tissue culture flask. If attachment does not occur under these conditions, we can conclude that either attachment is not required for metacyclogenesis in *T*. *brucei* or that overexpression of *Tb*RBP6 allows cells to bypass a step that is required in vivo. As discussed above, even if attachment is not mechanistically linked to differentiation of *T*. *brucei*, it is probably still important in the fly to boost parasite numbers in the salivary glands and prevent elimination of the parasites by blood feeding before they have differentiated to infectious forms.

In *T*. *cruzi*, where attachment is essential for differentiation to metacyclics, a putative RNA-binding protein has been described that is expressed exclusively in epimastigote and metacyclic forms [150]. Knockout of this protein, termed *Tc*ZC3H31, in epimastigotes prevents differentiation to the metacyclic form, while overexpression increases metacyclogenesis. It seems likely that *Tc*ZC3H31 is regulated by attachment and may in turn regulate a set of transcripts required for complete transformation to the metacyclic form. Another putative RNA-binding protein, *Tc*RBP35, was up-regulated in starvation conditions used to trigger metacyclogenesis [151].

Proteomics approaches in *T*. *cruzi* have also identified proteins associated with antioxidant defense, including glutamate dehydrogenase and dihydrolipoamide dehydrogenase, that are regulated and/or differentially modified during the transition from epimastigote to metacyclic [151–153]. Nutritional stress could alter cellular redox state leading to regulation of metabolic pathways including a shift from carbohydrate breakdown to metabolism of amino acids derived from hemoglobin [152–154]. Finally, attached *T*. *cruzi* cells are enriched in a major facilitator superfamily protein similar to the PAD proteins (protein associated with differentiation) described in *T*. *brucei* [155] as well as the putative FAZ component *Tc*GP72 [151,156,157].

In *P*. *confusum* and *C*. *fasciculata*, in vitro attachment was leveraged to compare gene expression profiles between swimming and attached cells [21,61]. Both revealed large-scale changes, indicating that these are in fact distinct developmental stages. For *P*. *confusum*, predicted dispersed gene family 1 (DGF-1) proteins were up-regulated in attached haptomonads compared to free-swimming promastigotes. These membrane proteins are best characterized in *T*. *cruzi* [158,159] and are secreted during the transition to the intracellular amastigote form. Interestingly, DGF-1 proteins have some similarities to integrins, which are found in hemidesmosomes and mediate cell-substrate attachment in other eukaryotes [160]. Free-living bodonids also express a family of surface molecules, some of which resemble DGF-1 while others resemble *Tc*GP72 [161].

Although *T*. *brucei* does not attach in vitro, single-cell RNA sequencing studies have provided gene expression profiles for parasites attached to the tsetse salivary gland [78,162,163]. Attached cells have profiles overlapping those of epimastigotes and metacyclics, suggesting they are an intermediate form and emphasizing the importance of attachment in this developmental transition [162]. As expected, surface proteins such as *Tb*BARPs and *Tb*MISP were expressed in attached cells. Consistent with the *T*. *cruzi* study mentioned above, several *Tb*PAD proteins were enriched in attached epimastigotes [163]. In multiple studies, the top epimastigote hits included a calpain-related protein (with some similarity to KIAP2 and 3 although not directly orthologous), a hypothetical protein, and an amino acid transporter [78,162,163]. Zinc finger proteins and putative RBPs *Tb*ZC3H11 and *Tb*ZC3H45 were found associated with differentiating pre-metacyclics. Perhaps these proteins are activated in attached cells and contribute to the establishment and maintenance of metacyclic gene expression profiles [78,162].

## Conclusions and future directions

Unlike hemidesmosome-attached cells found in tissues of multicellular organisms [164,165], attached parasites must either detach or produce a swimming cell through cell division in order to complete their life cycle. This, combined with the fact that the plaque itself is composed of novel or highly diverged components, makes trypanosomatid attachment a compelling model for the evolution of substrate attachment and related signaling. In addition, as a crucial stage in the life cycle of all pathogenic trypanosomatids, mechanistic insights into attachment may lead to novel transmission blocking strategies. Although technical limitations have previously precluded detailed molecular analysis of attached stages, new methods are being applied to multiple species both in vitro and in vivo to uncover the fundamental mechanisms underlying this process. A full description of attachment and detachment requires integrating knowledge from many fields of trypanosomatid biology that have been of intense research interest, including flagellum and cytoskeleton biology, environmental sensing, cell signaling, gene expression, surface proteins, and differentiation. Comparative studies will underscore the high degree of conservation between species while revealing how attachment has been adapted to facilitate the diverse life cycles of these parasites.
